# Dynamical Properties and Conceptual Interpretation of Latent Change Score Models

**DOI:** 10.3389/fpsyg.2021.696419

**Published:** 2021-07-29

**Authors:** Pablo F. Cáncer, Eduardo Estrada, Mar J. F. Ollero, Emilio Ferrer

**Affiliations:** ^1^Department of Social Psychology and Methodology, Autonomous University of Madrid, Madrid, Spain; ^2^Department of Psychology, University of California, Davis, Davis, CA, United States

**Keywords:** latent change score models, longitudinal data analysis, structural equation models, dynamic models, developmental trajectories, theory of change

## Abstract

Latent Change Score models (LCS) are a popular tool for the study of dynamics in longitudinal research. They represent processes in which the short-term dynamics have direct and indirect consequences on the long-term behavior of the system. However, this dual interpretation of the model parameters is usually overlooked in the literature, and researchers often find it difficult to see the connection between parameters and specific patterns of change. The goal of this paper is to provide a comprehensive examination of the meaning and interpretation of the parameters in LCS models. Importantly, we focus on their relation to the shape of the trajectories and explain how different specifications of the LCS model involve particular assumptions about the mechanisms of change. On a supplementary website, we present an interactive Shiny App that allows users to explore different sets of parameter values and examine their effects on the predicted trajectories. We also include fully explained code to estimate some of the most relevant specifications of the LCS model with the R-packages *lavaan* and *OpenMx*.

## Introduction

A Latent Change Score (LCS) model is a latent variable dynamic model for the analysis of processes that unfold over time. It was originally introduced by Hamagami and McArdle (2001) and McArdle (2001) and it stemmed from the need to incorporate a dynamic perspective into developmental research. In dynamical systems theory, development is defined as the continuous interaction of all the elements within a system as it unfolds over time (Thelen and Smith, 2006), and the observed changes are represented as a function of the previous states of the system (Voelkle et al., 2018). Consider, for example, embryonic development in smoking mothers, or cognitive reserve and memory declines in aging. In both scenarios, the multivariate repeated measures taken from the individuals are conceptualized as developing systems in which the changes are partially determined by some previous conditions. Initial fluctuations in the chemical composition of the amniotic fluid will alter the patterns of interaction between cells, which will affect the process of tissue differentiation and organ formation. Likewise, the genetic heritage, environmental stimulation, and developmental history of the elderly will determine the rate at which cognitive decline occurs.

To study the dynamic and multivariate nature of developmental phenomena such as those described above, statistical models are needed that provide a representation of the process where past events have future consequences, and the mechanisms of change can be continuously affected by internal and external influences. LCS models were developed for this purpose and are a flexible framework for examining dynamics in longitudinal research. In particular, they are typically applied to situations in which the interest is in characterizing both the mean changes as well as the covariances over time (as opposed to, say, auto-regressive models where the main focus are the covariances, cf., McNeish and Hamaker, 2020). As such, researchers have used different versions of LCS models to study change in a variety of constructs, including cognitive function and academic achievement in children (Peng and Kievit, 2020), pulmonary function and fluid intelligence in middle-aged and older adults (Finkel et al., 2013), or depressive symptoms and perceptual speed in late life (Bielak et al., 2011), among others. Despite this popularity, and the existence of excellent literature on how to specify the model and interpret some of its features (i.e., Ghisletta and McArdle, 2012; Kievit et al., 2018), several key aspects of LCS models remain misunderstood. In particular, the interpretation of some of the model dynamic parameters and their connection with theoretical mechanisms of change has not been addressed in a comprehensive way.

In this paper, we aim to provide a clear and accessible interpretation of the parameters in LCS models and their dynamical properties. First, we will provide a conceptual and mathematical description of the LCS model in connection with the dynamic systems literature. Second, we will clarify how LCS parameters can be interpreted to test hypotheses about both the short-term dynamics and the long-term development of a system. Third, we will extend this interpretation to bivariate LCS models and elaborate on the importance of including prediction errors at the latent level. Finally, we conclude with some examples of how the LCS model can be adapted to represent specific theoretical mechanisms of change. Furthermore, we provide an interactive online Shiny App to examine how the model parameters can be modified to express different patterns of change, as well as *lavaan* and *OpenMx* code for the estimation of LCS models (see https://marjfollero.github.io/LCSmodels/docs/index.html).

## Univariate Latent Change Score Models

Imagine a researcher who is interested in the development of reading abilities from childhood to early adulthood. Her goal is to study reading acquisition and performance during this developmental period and to examine whether the evolution of reading is related to other relevant variables, such as cognition, socio-economical status, or academic performance. For such purpose, she decides to use a latent change score approach, which is known to be a flexible and powerful model for the analysis of developmental data.

Because LCS models are built as structural equation models, our researcher can easily separate the true scores from their measurement error, so the change is studied at the latent level. Thus, her LCS model represents the observed state of reading performance *Y* for individual *i* at any given time *t* as a function of a latent initial level (*y*_0_), and the past history of changes up to that time (Δ*y*_*ik*_), plus some measurement error (McArdle and Hamagami, 2001; McArdle, 2001, 2009):

(1) $$Y_{i\left[t\right]}=y_{i,0}+\sum_{k=1}^{t}\text{Δ}y_{ik}+\varepsilon_{i\left[t\right]}$$

where *k* quantifies the number of discrete changes up to time *t*. Based on this general expression, changes in reading performance (Δ*y*) can be expressed through different mechanisms. In a dynamical system, the level of the variable at any given point in time *t* constitutes the initial conditions for the change up to *t* + 1. However, our construct of interest is often only one of multiple processes in constant interaction within a larger system, and thus the scores at *t* + 1 will only be partially determined by the scores at the previous occasion *t*. Based on this idea, the latent changes can be predicted by (i.e., regressed on) several determinants of change.

For simplicity, our fictional researcher decides to focus her analysis on reading performance only, leaving other variables aside for the time being. She opts for one of the most typical specifications for univariate systems, the so-called dual LCS model, in which changes (Δ*y*_*i*[_*_*t*_*_]_) are determined by: (1) the latent level of the process at the previous occasion (*y*_*i*[_*_*t*–1_*_]_), through a self-feedback regression parameter β, representing the strength of the association between the change and the latent level at the previous occasion and (2) an additive component representing a linear effect on the system, *y*_*a,i*_, exerting influence through a regression parameter α:

(2) $$\text{Δ}y_{i\left[t\right]}=\alpha \cdot y_{a,i}+\beta \cdot y_{i\left[t-1\right]}$$

**FIGURE 1:**
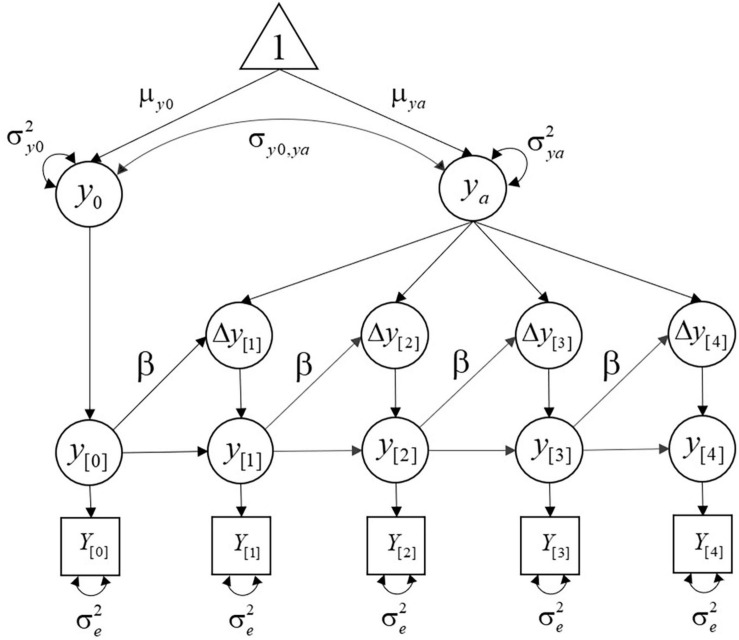
Univariate dual LCS model with five measurement occasions. Unnamed paths have weights of 1.

Because the coefficient α is usually fixed to 1, it will be omitted in the following sections (for other alternative specifications of change, see McArdle and Nesselroade, 2014; Hamagami and McArdle, 2018). Figure 1 illustrates a path diagram of the univariate dual LCS model with seven parameters: two means (μ*_y_*_0_ and μ*_*ya*_*), three variances (σ*_y_*_0_^2^, σ*_*ya*_*^2^, and σ_*e*_^2^), one covariance (σ*_y_*_0,_*_*ya*_*), and one self-feedback effect (β).

The LCS model in Figure 1 combines between- and within-individual information from the initial level of reading performance, the additive component, and the self-feedback to generate specific trajectories for each individual. Children are expected to exhibit differences in reading ability, not only at the first measurement occasion, but also during their development. For that reason, the researcher specified two sources of between-individual variability: (1) the initial level, which captures the mean μ*_y_*_0_ and variance σ*_y_*_0_^2^ in the latent level at the first measurement occasion and (2) the additive component, with mean μ*_*ya*_* and variance σ*_*ya*_*^2^. Between-individual differences in the trajectories are then determined by the combination of σ*_y_*_0_^2^ and σ*_*ya*_*^2^ over time. These two elements are usually allowed to correlate, with covariance σ*_y_*_0,_*_*ya*_*. Thus, if children with lower initial reading scores change more than those with higher initial reading scores, the correlation between the initial level and the additive component will be negative.

Once the latent initial level of reading performance for each individual is specified, the developmental trajectories are defined as a function of the additive component and the self-feedback (β). This means that all within-person variability at the latent level is determined by the change equation (Equation 2), and any fluctuation around the implied individual trajectories is considered measurement error (with variance σ_*e*_^2^). That is, LCS models estimate latent trajectories that approximate the observed data, and any difference between the predicted trajectories and the observed scores will be treated as measurement error. However, it is also possible to include an additional source of within-person variance in the form of prediction error to account for deviations from the expected changes (see the section “Introducing Uncertainties at the Latent Level: Stochastic LCS Models,” below, for a conceptual and mathematical description of this model specification).

Importantly, the researcher in our example specified a time-invariant additive component and self-feedback, reflecting a particular mechanism of change that governs the development of reading performance throughout the sampled time range. In other words, the constant amount of change added on each occasion, as well as the relation between the previous level of reading performance and the subsequent changes, are invariant over time. However, this does not necessarily have to be the case, as it depends on our hypothesis about the mechanism that generates the observed changes. For example, some theoretical frameworks include changes in the nature of a construct occurring at different stages, such as Piaget’s stages of child development (Piaget and Inhelder, 1969). Similarly, our researcher could be planning an intervention on dyslexic children with the aim of modifying some reading-related mechanisms of change during a treatment phase. If the mechanisms that bring about change are expected to change over different phases or stages, extensions of LCS models such as time-varying parameters (see Butner et al., 2021) or regime-switching (see Chow et al., 2013) may be more appropriate.

## Relation Between Lcs Parameters and Exponential Trajectories

When examining visually the development of reading, as well as other cognitive variables, our researcher realizes that they follow exponential trajectories of various shapes. After fitting univariate LCS models to these variables, she notices that the self-feedback and the additive component vary substantially from one variable to another, both in sign and magnitude. In fact, the differences in these parameters seem to be related to the shapes of the trajectories. But what is the connection between these components?

First, in order to better understand the relationship between the self-feedback, the additive component, and the specific shape of the resulting trajectory, it is useful to examine the relation between the univariate LCS model and the exponential function below:

(3) $$y\left(t\right)=y_{As}-\left(y_{As}-y\left(t_{0}\right)\right)\cdot e^{-r\cdot t}$$

**FIGURE 2:**
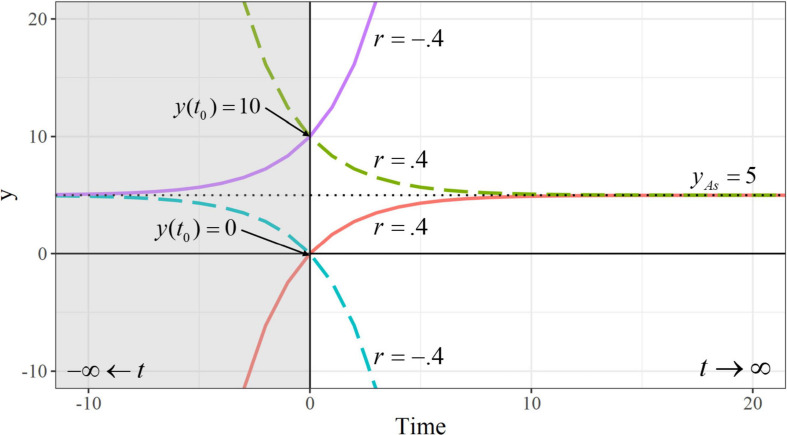
Exponential trajectories with varying values for the initial level *y*(*t*_0_) and the rate of change *r*. Note: Negative values of time are shaded. The asymptote is represented by a horizontal dotted line.

This expression looks very different from the LCS equations presented in the previous section, but we will clarify their relation shortly. Using Equation 3, our researcher can represent the level of a variable *y* at any given time *t* as a function of the asymptote of the trajectory *y*_*As*_, the initial level *y*(*t*_0_), and the rate of change *r* (i.e., rate at which the distance between those two components is reduced).^1^ Figure 2 illustrates four exponential trajectories derived from Equation 3 with varying values for *r* = {−0.4 and 4} and initial level *y*(*t*_0_) = {0 and 10}, and a fixed asymptote *y_As_* = 5.

The asymptote *y*_*As*_ represents a horizontal line, where the trajectory flattens out as time approaches −∞ or ∞, and the initial level *y*(*t*_0_) represents the intercept of the trajectory (i.e., the value of the variable *y* when *t* = 0). If *r* is positive, the variable *y* approaches its asymptotic level as *t*→∞, while growing (green-dashed line) or decaying (red line) without bound as *t*→−∞. In contrast, negative values of *r* indicate that *y* approaches its asymptotic level as *t*→−∞, while exhibiting an accelerated growth (purple line) or decay (blue-dashed line) when *t*→∞. Of course, the study of developmental processes only makes sense when time moves from the past into the future (i.e., when *t*→∞). Therefore, if the development of reading performance describes decelerated change over time, it will be defined by a positive *r*, which determines how fast the difference between the asymptote and the initial level (*y*_*As*_ – *y*(*t*_0_)) is reduced over time. In contrast, if it describes a pattern of accelerated change, it will be defined by a negative *r*, representing the speed at which the level of *y* moves away from the asymptote over time. In other words, the magnitude of *r* indicates the speed at which reading performance moves from the initial point to the asymptote (when *r* is positive), or away from the asymptote (when *r* is negative).

The dual LCS model represented in Equation 2 describes the changes in reading ability between measurement occasions, which are modeled as latent variables Δ*y*_*i*[_*_*t*_*_]_. In contrast, in the exponential function in Equation 3 we describe the level of reading ability *y*(*t*) on each occasion, instead of the changes in between. Both approaches, however, are very closely related, because Equation 2 is an approximation to the first derivative with respect to time of Equation 3. The derivative of Equation 3 results in a first order ordinary differential equation that provides the change in reading ability *dy*(*t*) as a function of the level *y*(*t*), for an infinitesimally brief time lag *dt* (Brown, 2007):

(4) $$\frac{dy\left(t\right)}{dt}=r\cdot y_{As}-r\cdot y\left(t\right)$$

By replacing *r*⋅ *y*_*As*_ with the additive component *y*_*a*_, and *r* with −β, this expression becomes:

(5) $$\frac{dy\left(t\right)}{dt}=y_{a}+\beta \cdot y\left(t\right)$$

which represents the continuous-time (CT) version of Equation 2. Note that the latent changes in Equation 2 are defined in discrete-time (DT) because they represent the change in reading performance Δ*y*_[_*_*t*_*_]_ for a specific time lag of Δ*t* = 1 (i.e., the left-hand side of Equation 2 could be specified as Δ*y*_*i*[_*_*t*_*_]_/1, but it is usually simplified by removing the denominator). In contrast, changes in Equation 5 are defined in CT, as they represent the change in reading ability *dy*(*t*) for an infinitesimally brief time lag *dt*^2^.

It is important to note that, because the additive component and self-feedback from Equation 5 are defined in CT, they are only equivalent to their counterparts in Equation 2 if they are first rescaled to a DT metric for a particular time lag Δ*t*. Nonetheless, the relation between the model parameters and the shape of the trajectory is the same regardless of the time metric and specific time interval. In this paper we focus on clarifying this relation. For detailed information on the correspondence between LCS-DT and LCS-CT models, see Voelkle et al. (2012), Voelkle and Oud (2015), and Estrada et al. (2020).

## Interpreting the LCS Parameters: Short-Term Dynamics and Long-Term Trajectories

In our previous description of the univariate LCS model, we defined the parameters in terms of the short-term dynamics of the system: the self-feedback indicated the extent to which the changes in reading performance *y* at time *t* were determined by the scores at the previous time *t* − 1, and the additive component was a constant amount added on each occasion. The relation between the univariate LCS model and the exponential function provides a new understanding of how these short-term changes can lead to different long-term developmental trajectories. In this section, we describe the type of trajectories that univariate LCS models can reproduce. Next, through the relation between Equations 2 and 5, we elaborate on the meaning of the LCS model parameters and the information they provide about change.

**FIGURE 3:**
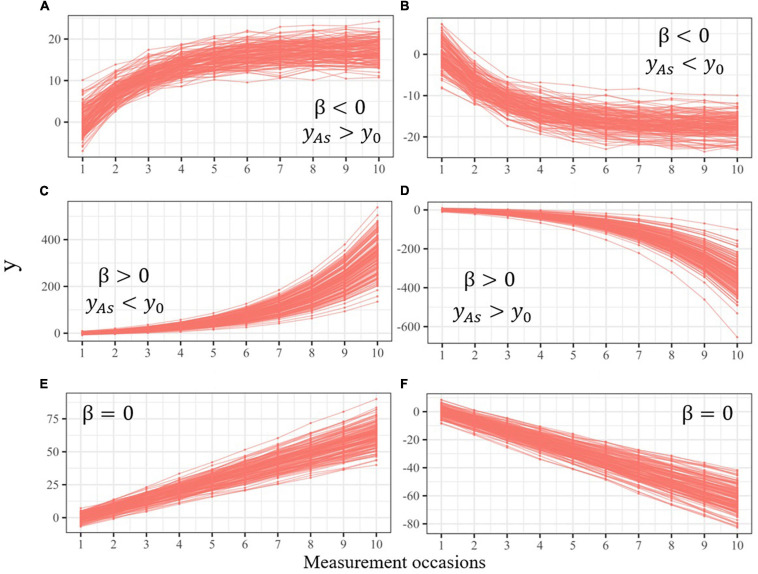
Individual implied trajectories from a univariate LCS model with self-feedbacks of varying sign. Self-feedbacks are negative **(A,B)**, positive **(C,D)**, or zero **(E,F)**, indicating decelerated, accelerated, or linear change, respectively.

Figure 3 illustrates the four patterns of non-linear change that the univariate LCS model can describe, and two additional patterns of linear change: A) decelerated growth (*y_As_* > *y*_0_ and β < 0); (B) decelerated decline (*y_As_* < *y*_0_ and β < 0); (C) accelerated growth (*y_As_* < *y*_0_ and β > 0); (D) accelerated decline (*y_As_* > *y*_0_ and β > 0); (E) linear growth (β = 0); and (F) linear decline (β = 0).^3^

Importantly, the LCS model is suitable for examining longitudinal data when the mean structure is non-stationary (i.e., the average scores are expected to grow or decline over time). One key, and often overlooked, aspect of the model is that it specifies the same mechanism of change (i.e., set of equations) for characterizing both the short-term dynamics and the long-term developmental trajectories (see Equation 2). This feature makes the LCS model fundamentally different from other models detaching the longitudinal mean change from the autoregressive process, such as Random Intercept Cross-Lagged Panel Model, or the Stable Trait Autoregressive Trait and State model (see, for example, Usami et al., 2019).

Specifying a common mechanism for both aspects of change is consistent with most theories of biological and psychological development across the lifespan. Some examples of such processes are age-related declines in cognitive function (Ritchie et al., 2015), reading and writing skills during childhood (Ahmed et al., 2014), volume changes in cortical structure in childhood and adolescence (Bengtsson et al., 2005), or physical functioning in aging (Sargent-Cox et al., 2012). Other psychological phenomena are expected to fluctuate around a stationary mean, such as state anxiety in adolescents during the academic year (Steinmayr et al., 2016), marital satisfaction in married couples (Hawrilenko et al., 2016), or the emotional evolution after a romantic breakup (Sbarra and Ferrer, 2006). If the changes in the mean structure are not considered important for the study, LCS models are not the best option. In that case, other dynamic models such as the Random Intercept Cross Lagged Panel Model (RI-CLPM) (see, Hamaker et al., 2015; Mulder and Hamaker, 2020; Zyphur et al., 2020) may be more appropriate.

### Self-Feedback: Decelerated and Accelerated Change

Based on the relation between Equations 4 and 5, the researcher in our example can now interpret the self-feedback parameter as a rate of change, which determines how fast the level of reading performance grows or decays with respect to the asymptote. For any initial level (i.e., at *t* = 0) and asymptote, larger negative (or positive) self-feedbacks will result in trajectories that approach (or move away from) the asymptote more quickly.

**FIGURE 4:**
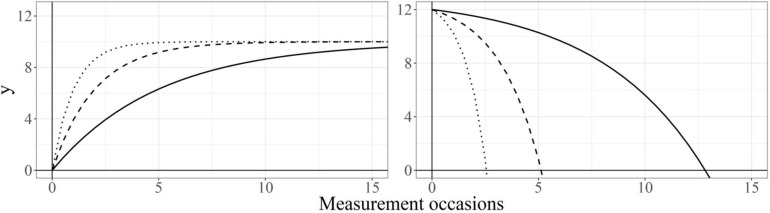
Grand-mean implied trajectories of decelerated growth and accelerated decay. Note: Values of self-feedback β = −0.2, −0.5, −1 **(left)** and β = 0.2, 0.5, 1 **(right)** are represented by solid, dashed, and dotted lines, respectively.

For example, suppose that the researcher is interested in the differences in reading performance among children from low, medium, and high socio-economical backgrounds (SEB). In particular, she hypothesizes that children with low SEB will experience a slower growth in, say, reading comprehension, and a slower decrease of reading mistakes compared to children with higher SEB. After fitting a LCS model to each group of children, she estimates the parameters and plots the latent trajectories. Figure 4 depicts the group-mean implied trajectories of reading comprehension (left panel) and reading mistakes (right panel) for each group of SEB.

In reading comprehension, the self-feedback parameters are −0.2, −0.5, and −1, for the groups with low, medium, and high SEB, respectively (left-panel of Figure 4). In its short-term interpretation, negative self-feedbacks mean that, across all groups, children with higher reading performance at *t* − 1 will experience smaller changes at time *t* compared to children with lower reading performance. If this pattern is consistent over time, all children will experience smaller subsequent changes as more time passes. This leads to long-term developmental trajectories in which rapid improvements during the early years are followed by a progressive deceleration. In this context, a less negative self-feedback for the low-SEB group (β = −0.2) is interpreted as a slower growth in reading comprehension with respect to the medium- and high-SEB groups (with β = −0.5 and β = −1, respectively), as the researcher hypothesized.

In reading mistakes, our researcher obtained self-feedbacks of 1, 0.5, and 0.2, for the groups with high, medium, and low SEB, respectively (right panel of Figure 4). In its short-term interpretation, positive self-feedbacks mean that individuals with few reading mistakes at time *t* − 1 will make even fewer mistakes at time *t*. If this pattern is maintained over time, all individuals will commit fewer subsequent mistakes as more time passes. This leads to trajectories in which reading mistakes become more rare with the passage of time, and more positive self-feedbacks will be interpreted as faster decays. In this context, a more positive self-feedback for the high-SEB group (β = 1) is interpreted as a faster reduction in reading mistakes with respect to the medium- and low-SEB groups (with β = 0.5 and β = 0.2, respectively).

Note that the researcher specified the self-feedback as a single fixed parameter, and therefore all the cases in each sample are assumed to have the same rate of change. In other words, all the children’s trajectories are assumed to move toward (or away from) their respective asymptotes at the same speed. This assumption may not be realistic in some scenarios, and some recent approaches allow including random effects in the self-feedback parameter (see Driver and Voelkle, 2018).

In empirical applications of the LCS model it is also possible to obtain self-feedback values of 0 (bottom panels of Figure 3), meaning that scores at time *t* are not affected by the previous scores at time *t* − 1. In this scenario, all the changes are explained by the additive component, which is interpreted as a slope, leading to linear trajectories where change is constant over time. This pattern of change has been found when measuring age-related declines in lifestyle activities (Small et al., 2012) and well-being (Gerstorf et al., 2007), cortical thickness from childhood to early adulthood (Estrada et al., 2019), or decreases in pulmonary function in aging (Finkel et al., 2013). Although non-linearities are frequent in dynamical systems, linear patterns of change are also possible. However, self-feedback parameters with values of 0 may also be obtained when the intervals between measurement occasions are too large to capture the short-term dynamics of the phenomenon under study (see Voelkle et al., 2012), or when the sampled time range is too short to capture the nonlinearities in the long-term trajectories.

### The Additive Component: A Source of Asymptotic Variance

Based on the mathematical equivalence between Equations 4 and 5, the additive component in the univariate LCS model can be expressed as:

(6) $$y_{a,i}=y_{As,i}\cdot \left(-\beta \right)$$

This relation reveals that the additive component contains information about the asymptotes of the individual trajectories. In fact, the variance σ*_*ya*_*^2^ captures between-individual variability in the asymptote (or maximum level) of these trajectories.

**FIGURE 5:**
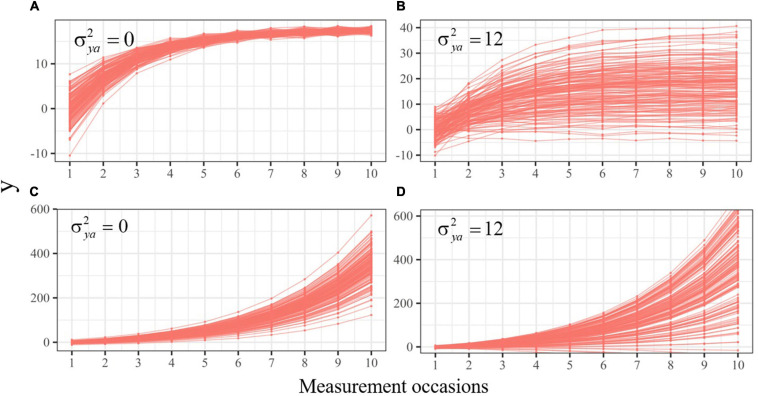
Individual implied trajectories of decelerated **(A,B)** and accelerated **(C,D)** change with values of 0 **(A,C)** and 12 **(B,D)** for the variance of the additive components. The variance in the initial latent state is 10 across all sets, and the value of β is constant between the cases in each set.

Importantly, when the reading researcher includes the variances of the initial level and additive component in her model (see Figure 1 and Equation 2), she is allowing individuals to have different baselines and tend toward different asymptotes. Also, she is assuming that *y*_0_ and *y*_*a*_ follow a bivariate normal distribution. Therefore, the scores in reading performance are expected to follow a normal distribution at the initial time point, at the asymptote, and at any time point in between. Figure 5 depicts four sets of individual trajectories representing four different measures of reading performance with σ*_y_*_0_^2^ = 10, fixed value for β, and varying values for the variances of the additive components. Let us suppose that these measures represent reading comprehension (panel A), fluency (panel B), phonetic coding (panel C), and reading decoding (panel D).

When the change is decelerated, the variance of *y* is reduced over time as it is repeatedly multiplied by the negative self-feedback parameter. In reading comprehension (Figure 5A), the additive component does not have a variance to compensate for this reduction (σ*_*ya*_*^2^ = 0), meaning that individual trajectories will converge toward the same point. At the beginning of the study (i.e., *t* = 0), the level of reading comprehension differs substantially between children (σ*_y_*_0_^2^ = 10), but eventually all will reach the same level. In fluency (Figure 5B) the observed trajectories are defined by a large variance in the additive component (σ*_*ya*_*^2^ = 12), implying greater dispersion of the asymptotes. On the other hand, the change in phonetic coding and reading decoding is accelerated (β > 0), implying that the variance of both processes is amplified over time, leading to trajectories that will spread indefinitely without bounds (Figures 5C,D). In this scenario, a larger variance in the additive component, such as σ*_*ya*_*^2^ = 12 in reading decoding, will amplify even more this dispersion.

The additive component also contains within-person information about the relative position of the asymptotes. For a fixed initial level and self-feedback, individuals with larger scores in the additive component will present asymptotes farther away from their initial level, implying larger growth or decline. It is often believed that positive (or negative) additive components lead to increasing (or decreasing) trajectories. However, this is not necessarily true. For example, both decelerated trajectories depicted in Figure 2 share the same asymptote *y*_*As*_ and rate of change *r* ( = −β), and therefore the value of the additive component ( = *y*_*As*_/−β) is the same whether the trajectory grows or decays. The same is true with both accelerated trajectories. In the univariate LCS model, growth or decay patterns depend on the relative position of the initial level and the asymptote. If the asymptote is above the initial level, negative (or positive) self-feedback parameters will lead to decelerated growths (or accelerated decays). Conversely, when the asymptote is below the initial level, negative (or positive) self-feedback parameters will lead to decelerated decays (or accelerated growths).

Note that Equation 6 illustrates a dependency between the additive component and self-feedback parameters, which explains the strong correlations between these components found in previous studies (see Jacobucci et al., 2019). This dependency implies that, if our researcher had misspecified one of the model parameters (e.g., she used a time-invariant self-feedback that is actually time-varying in the population), the misspecification would be compensated by the other parameter to reproduce the observed data, resulting in biased estimates (see Clark et al., 2018).

## Bivariate Latent Change Score Model: Interrelations Between Latent Processes

Most psychological theories represent developmental phenomena as the dynamic interaction between multiple processes over time. Aware of this, the researcher in our running example decides to examine the relations between reading and other cognitive variables. Based on previous literature (e.g., Ferrer et al., 2010), she hypothesizes that changes in children’s reading performance will be determined to some extent by cognitive ability level. For such purpose, she decides to use a bivariate dual LCS (BLCS) model, which allows examining the interrelations between two variables as they unfold over time. The specification of changes in this BLCS model (McArdle, 2001) can be represented as a bivariate extension of the dual LCS model from Equation 2 for reading performance (*x*) and cognitive function (*y*):

(7) $$\text{Δ}x_{i\left[t\right]}=x_{a,i}+\beta_{x}\cdot x_{i\left[t-1\right]}+\gamma_{x}\cdot y_{i\left[t-1\right]}$$

$$\text{Δ}y_{i\left[t\right]}=y_{a,i}+\beta_{y}\cdot y_{i\left[t-1\right]}+\gamma_{y}\cdot x_{i\left[t-1\right]}$$

In Equation 7, an additional determinant of change is included for each variable with respect to Equation 2: the parameters γ*_x_* and γ*_y_* are couplings that represent the cross-lagged influences from the level in each variable at time *t* − 1 to the change in the other variable at time *t*. Similar to self-feedbacks, positive (or negative) couplings mean that larger scores in one variable lead to larger (or smaller) subsequent changes in the other variable.

This specification of couplings leads to three possible types of developmental relations: (1) reading performance and cognitive ability are mutually interrelated over time (i.e., γ*_x_* ≠ 0 and γ*_y_* ≠ 0); (2) one process having a (positive or negative) impact on the changes in the other process, but not vice versa (e.g., γ*_x_* ≠ 0 and γ*_y_* = 0); and (3) both processes following dynamically independent courses over time (i.e., γ*_x_* = γ*_y_* = 0). This flexibility allows testing hypotheses about the directional effects between processes that develop differently across groups of individuals. For example, Ferrer et al. (2010) found positive and asymmetric couplings between reading and IQ in normative readers, meaning that both processes influenced each other over time, but also that IQ was a stronger predictor of changes in reading achievement than the other way around. These mutual interrelations, however, did not appear in dyslexic readers (as indicated by couplings not significantly different from zero), suggesting that reading and cognition develop more independently in these individuals.

Although couplings provide information on the temporal sequences between processes, they should not be interpreted as causal effects. Consider, for example, that our researcher finds a significant coupling indicating that higher levels of cognitive ability lead to higher levels of reading performance, but not vice versa (i.e., γ*_x_* > 0 and γ*_y_* = 0). This effect means that cognition temporally precedes, yet not necessarily causes, changes in reading performance. At best, one could argue that changes in cognition are not caused by previous reading levels, because the coupling is zero in that direction. Further causal claims, however, would require ruling out potential confounders and previous differences. Although significant couplings do not necessarily mean causation, if causation exists, it should be captured by significant couplings. Furthermore, coupling effects represent developmental sequences in which the predictions follow a temporal order. In regard to our researcher’s hypothesis, it would be reasonable to conclude that enhanced cognition predicts better reading performance, suggesting (but not proving) that improving cognitive abilities in children may lead to better reading performance with the passage of time.

### BLCS Models and Long-Term Developmental Changes

The short-term interpretation of additive components and self-feedbacks in the BLCS model is identical to that of the univariate LCS. However, when the couplings are non-zero, the non-linear behavior of the system is defined by the combinatorial effects of self-feedbacks and couplings over time, and the resulting long-term trajectories may not lead to a definite exponential form as in Equation 3. Consequently, the asymptote of the trajectories is determined by the interaction of the additive component, self-feedback, and coupling, implying that the relation *y_*As*_* = *y_*a*_*/(−β) does not necessarily hold in bivariate systems. In other words, the rate at which variables *x* and *y* move with respect to the asymptote^4^ is now determined by the continuous interaction between self-feedbacks and couplings, and the variance in the asymptotes (or maximum level) of the trajectories depends on the variances of both additive components.

**FIGURE 6:**
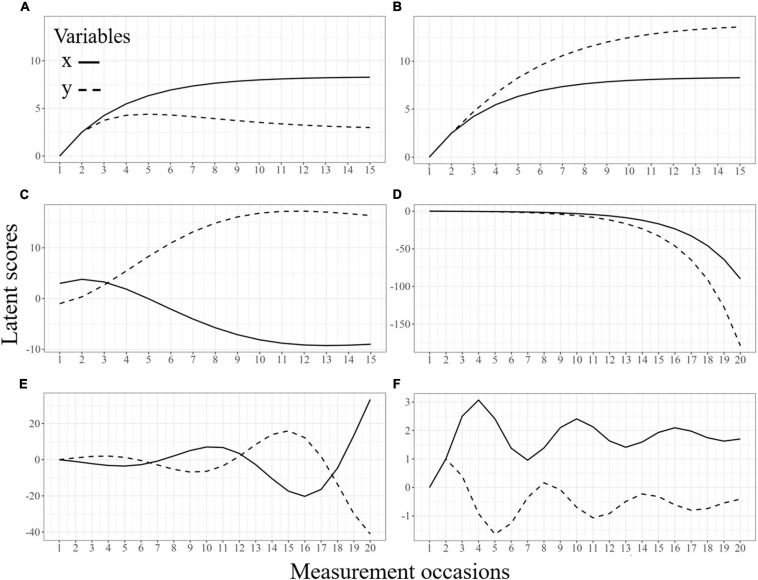
**(A–F)** Implied mean trajectories from a bivariate LCS model under various parameter values.

Figure 6 illustrates the type of non-linear trajectories that BLCS models can reproduce, defined by different values for the coupling parameters: (1) decelerated growth or decline (A, B, and C); (2) accelerated growth or decline (D); and (3) oscillatory behavior (E and F). Table 1 contains the time-lagged parameters leading to each of the six bivariate trajectories in Figure 6. Although not included in Figure 6, linear change patterns are also possible in BLCS models when the self-feedbacks and couplings are zero, meaning that both processes follow dynamically independent courses. Interested readers can enter the values from Table 1 in the supplementary Shiny App at https://marjfollero.shinyapps.io/BLCS_means/ to gain deeper understanding of how individual trajectories behave when the mechanism of change is defined by different coupling parameters.

**TABLE 1:** Generating time-lagged parameters of the bivariate trajectories in Figure 6.

	A	B	C	D	E	F
β_*x*_	−0.3	−0.3	0.3	0.3	0.9	–0.8
β_*y*_	−0.3	0.3	–0.7	0	–0.9	–0.3
γ_*x*_ (*y*→*x*)	−0.2	0.2	0.6	0.2	1.1	–0.8
γ_*y*_ (*x*→*y*)	0	0	–0.5	0.2	–1.1	0.8

Some phenomena consistent with trajectories in Figure 6 are the evolution of fluid reasoning and crystallized intelligence during the lifespan (A; e.g., McArdle et al., 2002), reading and verbal abilities during childhood (B; e.g., Ferrer et al., 2007), perceptual speed and depressive symptoms in older adulthood (C; e.g., Bielak et al., 2011), or memory and spatial abilities in aging (D; e.g., Finkel et al., 2007). The BLCS model can also describe more oscillatory behaviors through the combination of large self-feedbacks and couplings over time, as depicted in panels E and F. Importantly, these are mean trajectories, not equivalent to individual fluctuations around a stationary mean. In other words, they represent increases and decreases in the mean structure affecting all individuals in the sample at the same time, in contrast to other specification such as linear oscillators (Boker, 2001; Boker and Nesselroade, 2002; Boker et al., 2004; Chow et al., 2005).

One limitation of standard BLCS models is that irregular non-linear patterns and oscillatory behaviors can only be described when the changes are strongly dependent on previous states, as indicated by large couplings (see columns C, E, and F in Table 1). If this is not the case, BLCS models will not be able to accurately describe the dynamics of the system, and the implied trajectories will not reflect the true behavior of the latent processes. To overcome this limitation, Hamagami and McArdle (2018) proposed including an additional additive component with curvilinear effects for each process, allowing for irregularities in the non-linear trajectories.

## Introducing Uncertainties at the Latent Level: Stochastic LCS Models

Let us return to our example on children’s reading. In the LCS models described in the previous sections, latent changes were fully accounted for by the previous state of the latent process (or processes) and the additive component, without considering any prediction error. This specification is based on the assumption that, once the general mechanism of change is known, it is possible to predict perfectly the state of the system at any given point in time. In other words, it assumes that change is completely determined by the components of Equation 2 (i.e., deterministic change).

In empirical applications, however, individuals are often exposed to unobserved events that were not present in the initial state of the system, and whose influence can disrupt their change patterns over time. Suppose that our researcher has taken yearly measurements of children’s reading and academic performance. Some individuals in the sample may experience unpredictable events affecting (either positively or negatively) their cognitive functioning, such as starting private tutoring, the divorce of the parents, or a surgical operation leading to the loss of academic activity. The deterministic LCS models from previous sections do not account for the impact of such random events and assume that all the deviations from the predicted changes are measurement error. However, unlike measurement errors, these unpredictable events have an effect on the latent process. Moreover, their influence is not only limited to a specific point in time. Instead, they persist in later states of the system, potentially leading to deviations from the expected trajectories.

If our researcher believes that the developmental trajectories of reading and academic performance may be affected by unpredictable events or “random shocks”, she can account for those by including prediction error terms at the latent level in Equation 6:

(8) $$\text{Δ}x_{i\left[t\right]}=x_{a,i}+\beta_{x}\cdot x_{i\left[t-1\right]}+\gamma_{x}\cdot y_{i\left[t-1\right]}+d_{x,i}$$

$$\text{Δ}y_{i\left[t\right]}=y_{a,i}+\beta_{y}\cdot y_{i\left[t-1\right]}+\gamma_{y}\cdot x_{i\left[t-1\right]}+d_{y,i}$$

**FIGURE 7:**
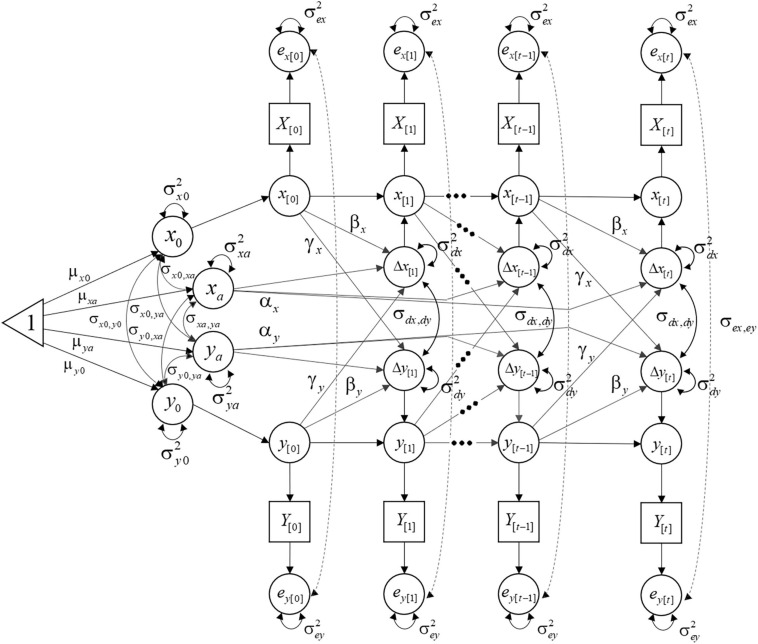
Path diagram of a stochastic BLCS model.

This expression describes a stochastic BLCS model. Here, *d*_*x*_ and *d*_*y*_ are random variables with mean 0, variances σ*_*dx*_*^2^ and σ*_*dy*_*^2^, and covariance σ_*dx, dy*_. These three additional parameters are usually fixed to be equal across time points (i.e., stationary or time-invariant). Figure 7 illustrates a path diagram of the stochastic BLCS model with 24 parameters.

**FIGURE 8:**
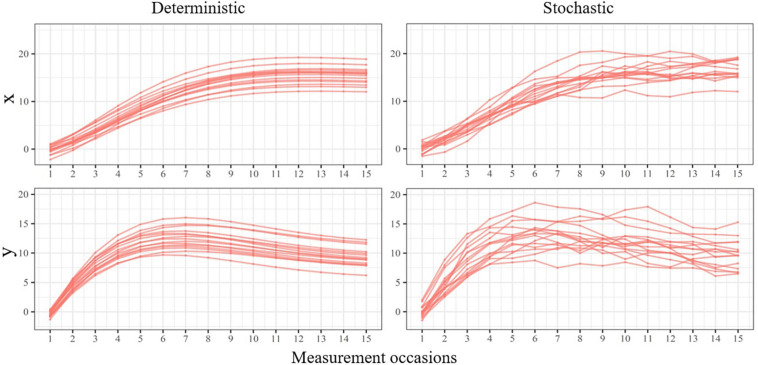
Individual implied trajectories from a deterministic **(left)** and stochastic **(right)** BLCS model.

Despite being specified as time-specific regression residuals, the deviations produced by these prediction errors are carried over through the self-feedback and coupling parameters to later states of the system. Because their effect in the latent process may be relevant, they are not considered merely “errors”. Instead, they are often termed innovations, dynamic errors, or dynamic fluctuations (Oud and Delsing, 2010; Schuurman et al., 2015; Voelkle et al., 2018). Figure 8 depicts individual latent trajectories from a deterministic (left) and a stochastic (right) BLCS model. Conceptually, this additional source of variance captures within-individual variability due to factors affecting the latent trajectories that cannot be explicitly included in the model as covariates, either because they are not known, cannot be measured, or any other reason. Such external factors act like random noise or random shocks and can affect each individual differently.

To date, the deterministic specification of the BLCS model has been prevalent in the psychology literature, probably because the simultaneous estimation of measurement errors and innovations tends to produce improper solutions and convergence errors under some conditions in other dynamic models, such as the RI-CLPM (see Hamaker et al., 2015; Lüdtke et al., 2018). However, a recent study has shown that, under appropriate sampling conditions, the stochastic BLCS model is able to capture the dynamics of the system and properly distinguish all sources of variance (Cáncer and Estrada, 2021). When the impact of innovations is expected at the latent level, the stochastic BLCS model allows a more realistic and accurate representation of dynamic systems.

## Closing Remarks: Additional Specifications of LCS Models

In the previous sections, we described how a developmental researcher reproduced a wide variety of change patterns using a dual LCS model (McArdle, 2001). However, other specifications are also possible—and may be more reasonable—depending on one’s theory of change. For example, given a particular hypothesis, the loadings of the additive components on the latent changes can be freed (instead of fixed to 1) to account for different amounts of baseline change at each repeated occasion (see the “triple change score model,” in McArdle and Nesselroade, 2014). Similarly, if the processes under study are expected to show quadratic curves in the long run, it would be possible to include an additional additive component with rescaled loadings representing quadratic change (see Hamagami and McArdle, 2018). These alternative specifications could potentially help the reading researcher in our example to obtain accurate information about change in phenomena describing more complex patterns (e.g., Figures 6C,E,F).

In a similar vein, hypotheses about acceleration rates (changes of changes) could be implemented by modeling the difference between adjacent latent changes as an additional latent variable (i.e., Δ*y*_*i*[_*_*t*_*_]_ −Δ*y*_*i*[_*_*t*_*_–1]_ = ΔΔ*y*_*i*[_*_*t*_*_]_). This new variable could then be regressed on the previous state *y*_[_*_*t*_*_–1]_ to represent a rate of acceleration (see Malone et al., 2004; Ferrer et al., 2007, for examples). In our example, the researcher could use this model specification to examine acceleration or deceleration in the developmental changes in reading, and test if these changes in speed can be explained by other variables. Likewise, the coupling effects in BLCS models can be re-specified to test different hypotheses of change. For example, we may hypothesize that changes in cognitive function temporally precede changes in reading performance, as indicated by significant change-to-change couplings (e.g., Δ*y*_*i*[_*_*t–*_*_1]_ →Δ*x*_*i*[_*_*t*_*_]_; see Grimm et al., 2012). It may also be theoretically relevant to consider the effect of the state of reading performance at *t* − 2 on the latent changes of the other variable at time *t*, as indicated by a significant 2-lag coupling. Importantly, all these specifications can be extended to include multiple indicators in the measurement structure of the model. For example, reading performance could be, at each time point, a composite of several measures, such as comprehension, fluency, and decoding. Although including multiple indicators does not provide additional information about the mechanisms of change, it ensures a more accurate representation of the latent constructs if they can be assumed to be invariant over time (for details on multiple indicators and longitudinal invariance in LCS models, see Ferrer et al., 2008).

In summary, LCS models are a general class of models that can be flexibly adapted to express a wide variety of hypotheses of change. The common feature of all these specifications is the use of time-sequential dynamics between latent constructs to simultaneously explain both the covariance structure and the changes in the means over time. This distinctive property is what differentiates LCS models from other frameworks, such as cross-lagged panel models (CLPM), which do not focus on growth or decline patterns, or latent growth curve models (LGCM), which ignore the time-lagged dynamics. Despite these differences, all these approaches are mathematically related, and both CLPMs and LGCMs can be obtained through re-specifications of the LCS model parameters (see Usami et al., 2015, 2019; Serang et al., 2019).

## Conclusion

Latent Change Score models are a flexible and powerful framework for studying dynamics in developmental processes. In this paper, we aimed to clarify the interpretation of the model parameters and describe their dynamical properties. In particular, we mapped these parameters onto theoretical mechanisms of change and explained their meaning in terms of both the short-term dynamics and the shape of the long-term trajectories. We hope the explanations provided in this paper, as well as the interactive Shiny Apps and R code included in the supplementary website, will help researchers in future applications of LCS models.

## Data Availability Statement

The original contributions presented in the study are included in the article/Supplementary Material, further inquiries can be directed to the corresponding author.

## Author Contributions

PC, EE, and EF conceived the structure and contents of the manuscript. PC wrote the first draft of the manuscript. MO designed the website. All authors reviewed, edited, and approved the final manuscript.

## Conflict of Interest

The authors declare that the research was conducted in the absence of any commercial or financial relationships that could be construed as a potential conflict of interest.

## Publisher’s Note

All claims expressed in this article are solely those of the authors and do not necessarily represent those of their affiliated organizations, or those of the publisher, the editors and the reviewers. Any product that may be evaluated in this article, or claim that may be made by its manufacturer, is not guaranteed or endorsed by the publisher.

## Footnotes

1 Interestingly, Equation 3 can be implemented as a latent growth curve model (LGC) to describe exponential growth. Serang et al. (2019) demonstrated that both LGC and LCS models are mathematically equivalent in certain scenarios. In such scenarios, both models provide identical information about growth, asymptotes, and rates of change.

2 The right hand of Equations 2 and 5 differs in the specification of the previous latent level of reading ability. For a time lag of 1 (DT), the previous level is represented as *y*(*t* − 1), whereas for an infinitesimally brief time lag *k* (CT), there is no difference between *y*(*t*) and *y*(*t*-*k*), and thus it is represented as *y*(*t*). Also note that, to describe change over time, we are using the Greek letter Δ in DT, and the Latin letter *d* in CT.

3 When the rate of change *r* is 0, Equation 3 describes a horizontal line without slope, and its derivative takes a value of 0. However, when the self-feedback β is 0 in a LCS model, linear trajectories can still be described, with the additive component acting as a slope. This occurs because the relation *r*⋅ *y*_*As*_ = *y*_*a*_ is only meaningful when the model is dynamic (i.e., future states of the system are determined by past states). In a LCS model, if β is exactly 0, growth (or decline) will be determined by a positive (or negative) additive component.

4 In a BLCS model, the implied trajectories do not necessarily have an asymptote. For example, a trajectory that accelerates when *t*→−∞, can also accelerate when *t*→∞ if it receives a nonzero coupling effect from another accelerated process.
